# Phototrophic N_2_ and CO_2_ Fixation Using a *Rhodopseudomonas palustris*-H_2_ Mediated Electrochemical System With Infrared Photons

**DOI:** 10.3389/fmicb.2019.01817

**Published:** 2019-08-14

**Authors:** Mathangi Soundararajan, Rhesa Ledbetter, Paul Kusuma, Shuyang Zhen, Paul Ludden, Bruce Bugbee, Scott A. Ensign, Lance C. Seefeldt

**Affiliations:** ^1^Department of Chemistry and Biochemistry, Utah State University, Logan, UT, United States; ^2^Department of Biological Sciences, Idaho State University, Pocatello, ID, United States; ^3^Department of Plant, Soils and Climate, Utah State University, Logan, UT, United States; ^4^Department of Biology, Southern Methodist University, Dallas, TX, United States

**Keywords:** microbial electrocatalysis, *in situ* fertilizer, bioelectrochemical nitrogen reduction, Haber–Bosch, bioelectrosynthesis, bioelectrochemical carbon dioxide reduction

## Abstract

A promising approach for the synthesis of high value reduced compounds is to couple bacteria to the cathode of an electrochemical cell, with delivery of electrons from the electrode driving reductive biosynthesis in the bacteria. Such systems have been used to reduce CO_2_ to acetate and other C-based compounds. Here, we report an electrosynthetic system that couples a diazotrophic, photoautotrophic bacterium, *Rhodopseudomonas palustris* TIE-1, to the cathode of an electrochemical cell through the mediator H_2_ that allows reductive capture of both CO_2_ and N_2_ with all of the energy coming from the electrode and infrared (IR) photons. *R. palustris* TIE-1 was shown to utilize a narrow band of IR radiation centered around 850 nm to support growth under both photoheterotrophic, non-diazotrophic and photoautotrophic, diazotrophic conditions with growth rates similar to those achieved using broad spectrum incandescent light. The bacteria were also successfully cultured in the cathodic compartment of an electrochemical cell with the sole source of electrons coming from electrochemically generated H_2_, supporting reduction of both CO_2_ and N_2_ using 850 nm photons as an energy source. Growth rates were similar to non-electrochemical conditions, revealing that the electrochemical system can fully support bacterial growth. Faradaic efficiencies for N_2_ and CO_2_ reduction were 8.5 and 47%, respectively. These results demonstrate that a microbial-electrode hybrid system can be used to achieve reduction and capture of both CO_2_ and N_2_ using low energy IR radiation and electrons provided by an electrode.

## Introduction

Coupling microbes to electrodes is a frontier area for specialty chemical production. This approach combines the advantages of a sustainable and efficient source of electrons with the varied and designable metabolism of microbes. In microbial fuel cells, oxidation of reduced carbon compounds (such as found in the wastewater or the environment) by bacteria coupled to an electrode (anode) results in a flow of electrons to the anode, either directly or indirectly through mediators, providing a usable electric current (Schröder et al., 2015). In the other direction, electrons delivered by an electrode (cathode), either directly or through mediators, can be used to drive the biosynthetic machinery of a microbial cell for the synthesis of compounds of interest (microbial electrosynthesis) (Rabaey and Rozendal, 2010; Schröder et al., 2015). Mediators commonly used to shuttle electrons between the bacteria and the electrode in microbial electrochemical systems include H_2_, formate, certain dyes (e.g., neutral red, methyl viologen) and other organic compounds (e.g., hydroquinone, anthraquinone-2,6-disulfonate) (Liu et al., 2018).

A number of microbial electrosynthetic systems have been used to fix CO_2_ (autotrophy) and upgrade the C to an array of value added compounds such as acetate, precursors for polymers, and precursors for pharmaceuticals (Rabaey and Rozendal, 2010; Liu et al., 2015). While these bacteria can obtain all C from CO_2_ reduction, a source of reduced N is also required to sustain life. Nitrogen is abundant in the Earth’s atmosphere as dinitrogen (N_2_), but accessing it requires energy intensive N_2_ fixation. This can be achieved by the industrial Haber–Bosch reaction for reduction of N_2_ using H_2_ over Fe based catalysts, which is efficient, but dependent on fossil fuels (Erisman et al., 2015). Alternatively, a number of bacteria and archaea contain nitrogenase, the enzyme that catalyzes the ATP-dependent reduction of N_2_ to ammonia (NH_3_) according to the minimal reaction stoichiometry for the Mo-dependent enzyme of:

N+28 H++16 MgATP+8 e-

  →2 NH+3H+216 MgADP+16 Pi

Nitrogenase requires a minimum of 16 ATP for each N_2_ reduced. For many nitrogen fixing bacteria, this energy comes from the consumption of reduced carbon compounds. However, this energy can come exclusively from light for nitrogen fixing, phototrophic bacteria.

Given the absolute need for fixed N to support bioelectrosynthesis and the high energy demand for conversion of N_2_ to fixed N, there would be value in developing microbial-electrode systems that could achieve both CO_2_ and N_2_ fixation. An earlier report showed that a complex microbial community from the environment could support both CO_2_ and N_2_ fixation driven by an electrode (Rago et al., 2019). While such a complex system has advantages, it does not easily work for production of desirable reduced N and C compounds since there could be symbiotic relationships within the community where the compounds produced by one organism may be used by another, and vice versa. In another system, the bacterium *Xanthobacter autotrophicus* could be grown on electrochemically produced H_2_ to achieve both CO_2_ and N_2_ fixation (Liu et al., 2017). Since *Xanthobacter* is not a phototroph, electrons abstracted from H_2_ by hydrogenase were required for both biosynthesis (e.g., CO_2_ reduction and N_2_ reduction) as well as energy generation via the electron transport chain. As a result, the currents needed to drive CO_2_ and N_2_ fixation were high (10–12 mA for a 100 mL reactor), since both processes (reductive biosynthesis and energy generation) consume electrons. The use of photoautotrophic N_2_ fixing bacteria in an electrosynthetic system could provide an energetic advantage since light energy captured by the bacteria could provide the energy needed to drive both CO_2_ and N_2_ fixation while the electrons abstracted from H_2_ will be used for biosynthesis among other processes.

Further, there would be value in utilizing very low energy (long wavelength, greater than 750 nm) photons to support such a system. For example, plants absorb radiation minimally beyond 750 nm (McCree, 1971; Noda et al., 2014; Nelson and Bugbee, 2015), and thus these longer wavelength photons are not used in intensive food production scenarios. In conditions where plants and the bacteria would have to be grown in the same place (e.g., indoor food production, on deep space missions, etc.) and sunlight is the most efficient light source (in terms of both energy and economy), making maximal use of wavelengths available is critical. Fiber optics can be used effectively to capture and transmit radiation between 400 to 800 nm with much higher energy efficiencies than artificial lighting. Since light (or energy source) could very well limit the growth of both plants and the bacteria, separating wavelengths that may be used for either process would be an added advantage. Earlier studies have indicated that some phototrophic bacteria can utilize these longer wavelengths of electromagnetic radiation (Kuo et al., 2012; Zhou et al., 2014; Qi et al., 2017; Xiao, 2017).

Here, a system is reported that couples the phototrophic bacteria *Rhodopseudomonas palustris* TIE-1 to an electrode through the mediator H_2_ and demonstrates bioelectrosynthetic N_2_ and CO_2_ fixation supported by low energy infrared (IR) photons.

## Materials and Methods

### Culture Media

*Rhodopseudomonas palustris* TIE-1 cells were used for all our experiments because the type strain CGA009 does not have a functional uptake hydrogenase. The bacteria were grown in a defined mineral medium containing 12.5 mM of Na_2_HPO_4_ and 12.5 mM of KH_2_PO_4_ as the buffer components, 0.002 mg mL^–1^ of *p*-aminobenzoic acid and 0.1% (v/v) final concentration of the concentrated base solution [stock solution containing the following components (concentration in mM): nitrilotriacetic acid (105), MgSO_4_ (240), CaCl_2_.2H_2_O (45), (NH_4_)_6_Mo_7_O_24_.4H_2_O (0.015), FeSO_4_.7H_2_O (2.51), EDTA (0.855), ZnSO_4_.H_2_O (3.808), MnSO_4_.H_2_O (0.911), CuSO_4_.5H_2_O (0.157), Co(NO_3_)_2_.6H_2_O (0.086), Na_2_B_4_O_7_.10H_2_O (0.046)] as described before (Kim and Harwood, 1991). The medium was also supplemented with 200 mM NaCl, 1 μM NiSO_4_, and 0.5% (v/v) final concentration of Wolfe’s vitamins stock solution [stock solution containing (in g L^–1^): *p*-aminobenzoic acid (0.005), folic acid (0.002), lipoic acid (0.005), riboflavin (0.005), thiamine (0.005), nicotinic acid (0.005), pyridoxamine (0.01), pantothenic acid (0.005), cobalamin (0.0001), biotin (0.002)]. The 200 mM NaCl was added in order to improve the electrical conductivity of the medium and was included in all other growths for consistency. Further, 1 μM NiSO_4_ was added as a supplement when hydrogen was provided as the electron donor since the uptake hydrogenase of *R. palustris* requires Ni as a cofactor.

For growing the cells photoautotrophically, but in the presence of fixed nitrogen, photosynthetic medium (PM) was prepared as described above, but with 0.1% (NH_4_)_2_SO_4_ as the nitrogen source and 30 mM NaHCO_3_ as the carbon source added after degassing the medium. Photoheterotrophic growth medium was the same as PM medium with 20 mM sodium acetate as the carbon source. For photoautotrophic, diazotrophic growth, nitrogen-fixing (NF) medium was prepared as described above and 30 mM NaHCO_3_ was added after degassing of medium. The pH of the medium was maintained at ∼6.8–7.0, unless otherwise specified.

### Measurement of *R. palustris* TIE-1 Whole Cell Absorbance and Leaf Absorption Spectra

*Rhodopseudomonas palustris* TIE-1 whole cell absorbance spectra were measured using a Cary 50 UV-visible spectrophotometer (Varian Instruments, CA, United States). Bacteria grown with either incandescent or IR light were pelleted and either used immediately or stored at −20°C until measurement. The cells were resuspended in NF medium until the absorbances at 660 nm were approximately 0.21. An empty cuvette was used to blank the instrument and the absorbance spectra were measured with a scan step of 1 nm and a scan rate of 250 nm min^–1^.

Soybean was used to obtain a percent absorption spectrum of a single leaf. Leaf transmission and reflectance measurements were made with halogen lamps using a spectroradiometer (Apogee Instruments, Model PS-200, Logan, UT, United States). Measurements were made from 400 to 850 nm at 1 nm intervals. Transmission was measured through the leaf 90° from the abaxial side. Reflectance was measured from the adaxial side over black felt (a highly absorbent material) so that only reflectance, and not trans-flectance, was measured. Absorptance was calculated as 1 − reflectance − transmission.

### Growth Conditions

#### Photoheterotrophic Growths to Analyze Effect of Wavelength on Growth of *R. palustris* TIE-1

Glass vials with photoheterotrophic growth medium (with acetate and ammonium sulfate) were prepared and degassed by sparging argon gas through the medium for 30 min and the headspace for 15 min. The vials were sealed using rubber stoppers, and autoclaved prior to inoculation. After cooling, the headspace was again sparged with argon gas for at least 10 min and equilibrated to 1 atm using a syringe. Then, a volume of H_2_ gas equal to the headspace volume of the vial was added to obtain a headspace gas composition of 1:1 of Ar:H_2_, at a pressure of 2 atm. NaHCO_3_ was added to a final concentration of 30 mM. *R. palustris* TIE-1 cells grown photoheterotrophically with a 60 W incandescent bulb was used as the inoculum. The cells were first pelleted and the pellets were washed twice with NF medium, to remove all traces of previous media and extracellular components prior to inoculation.

The cultures were then grown at room temperature (20–25°C) with light emitting diodes (LED) with peak wavelengths of 665 nm (Fluence Bioengineering, Model RAY22 custom spectra LED, TX, United States), 735 nm (Fluence Bioengineering, Model RAY22 custom spectra LED, TX, United States) and 850 nm (bought from Amazon.com), or broad spectrum cool white 6500 K LED (Fluence Bioengineering, Model RAY22 custom spectra LED, TX, United States). Cardboard boxes were used to exclude ambient light, and care was taken to minimize ambient light exposure while sampling. Light-source distances were adjusted to provide about 200 μmol photons m^–2^ s^–1^ to the culture vials. The cultures were not stirred or shaken at any point during the experiment except during sampling. The cultures were sampled using a disposable syringe that was made anaerobic by purging with argon gas three times prior to sampling, and the optical densities were measured using a Cary 50 UV-visible spectrophotometer (Varian Instruments, CA, United States) approximately every 4 h. All growths were performed in duplicates and the error bars represent standard deviations of the optical density measurements. Detailed description of the calculation of doubling times and specific growth rate are provided in the Supplementary Material.

#### Photoautotrophic, Diazotrophic Growth of *R. palustris* TIE-1 Using Incandescent or IR Photons

Photoautotrophic, diazotrophic growths were performed in glass vials. The NF medium was added to glass vials, and degassed by bubbling N_2_ gas through the medium for ∼20 min, and ∼10 min through the headspace. The vials were sealed with rubber stoppers and autoclaved. After cooling, the headspace was bubbled for another 20 min with N_2_ gas and equilibrated to 1 atm pressure. Then a volume of H_2_ gas equal to the volume of the headspace was added to obtain a headspace composition of 1:1 of N_2_:H_2_, at a final pressure of 2 atm. NaHCO_3_ was added for a final concentration of 30 mM. *R. palustris* TIE-1 cells grown under photoautotrophic, diazotrophic conditions with incandescent or IR light were used as inocula for the incandescent and IR growth conditions, respectively. The cells were pelleted and resuspended in NF medium prior to inoculation into the cultures.

The cultures were then grown at room temperature (20–25°C) with 60 W incandescent light or IR LED (with a peak of 850 nm). Cardboard boxes were again used to exclude ambient light. The light conditions were not standardized for the photon flux density since it was not possible to obtain a full spectrum of the incandescent light that typically extends to well beyond 1000 nm. Also, due to the very broad spectrum of the incandescent light, it would not be possible to identify which of the photons provided would be photosynthetically useful, and hence any measurement of the photon flux density would not be relevant to understanding its effects on the growth of the bacteria. The cultures were sampled at ∼24 h intervals as described above for photoheterotrophic growth and all growths were performed in duplicates.

### Growth in the Hybrid System

A microbial fuel cell (Adams and Chittenden Scientific glass, Berkeley, CA, United States) was used for bacterial growth with electrochemically produced H_2_. It was a modified H-cell with 150 mL cathodic and anodic compartments connected by half-inch glass flanges with corresponding seals and wraparound knuckle clamps. There were also two sampling ports on each compartment to sample either the media or the headspace. After autoclaving the glassware, a Nafion 117 cation exchange membrane was sandwiched between the flanges to separate the anodic and cathodic halves. Rubber stoppers were used to seal the sampling ports on the sides of the half cells as well as the top. The anode and cathode were made of 16-gauge platinum wires (∼3 cm) extended with copper wires. Electrodes were cleaned prior to each experiment by soaking in 5% HCl for 5 min and then wiping with ethanol. 100 mL of the autoclaved growth medium was used as the electrolyte in both the anode and the cathode compartments (NF medium for N_2_- and CO_2_-fixing growth and PM medium for the CO_2_-fixing growth). The electrolyte was made anaerobic by bubbling Ar or N_2_ gas through the anolyte and catholyte for 30 min and the headspace for 15 min. NaHCO_3_ was added at a final concentration of 30 mM after the medium was degassed. The electrolyte was constantly sparged with an 80:20 mixture of N_2_:CO_2_ gas in order to maintain anaerobicity, provide N_2_ and CO_2_ for bacterial growth as well as to maintain the pH at ∼7.5. *R. palustris* TIE-1 cells grown photoheterotrophically with 60 W incandescent or halogen light were used as the inoculum. The cells were pelleted, washed three times with NF medium, resuspended in 1 mL of degassed catholyte prior to inoculation and transferred to the cathodic compartment.

Electrolysis was performed at a constant potential difference of −3 V vs. the counter electrode (around −1.4 V vs. SHE) using a MultiEmStat (PalmSens BV, Netherlands) potentiostat to enable hydrogen evolution. This potential was chosen in order to achieve the currents necessary to support CO_2_ and N_2_ reduction. The cell was maintained at constant potential since the potentiostat did not have the capability to maintain constant current. An 850 nm LED light source (bought from Amazon.com) was used as the light source. The pH was checked regularly using pH strips and additional CO_2_ gas was sparged through the catholyte to reduce it to ∼7.5 if it was found to be ∼8.0 or above. The cultures were maintained at room temperature (20–25°C). Under CO_2_-fixing conditions, the cultures were not mixed at any time except during sampling. Additional 850 nm light was provided for the N_2_-fixing growth in the form of an additional, smaller IR LED (bought from Amazon.com), and the electrolyte was stirred. In our system, we found that this was important to support N_2_-fixation. We observed modification of the electrode surface during the course of the experiment which probably contributed to the slowly decreasing current over time (Supplementary Figure 1).

Samples were obtained once a day using disposable syringes purged with Ar or N_2_ gas as described for photoheterotrophic growths, through sampling ports on the sides of electrolysis cell. The optical density measurements were obtained as described for the photoheterotrophic growth. All growths were performed in duplicates.

### Measurement of Spectroradiometric Traces of Light Sources

The spectra of the lights were obtained using a spectroradiometer (Apogee Instruments, Models PS-100 and PS-200, Logan, UT, United States). Spectral measurements were made at the same distance that the light sources were placed from the culture vials for the photoheterotrophic growths so that the area under the curve could be integrated to calculate the photon flux density received by each culture. The area was integrated using the software IgorPro 6 (Wavemetrics, Portland, OR, United States).

## Results

### Absorbance Spectra of *R. palustris* TIE-1 and of Plant Leaves

**FIGURE 1:**
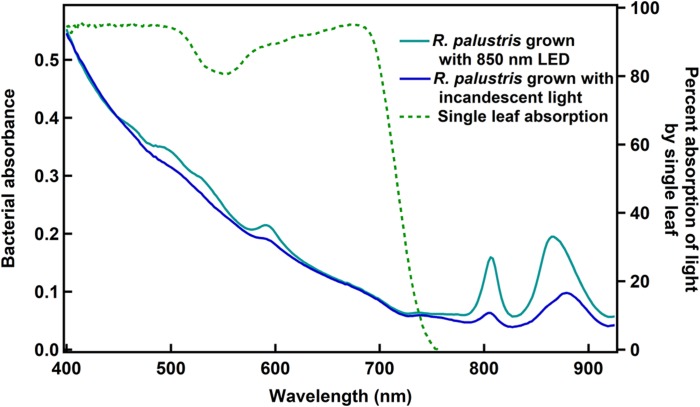
Whole cell absorbance spectra of *R. palustris* TIE-1 and the absorption spectrum of a single leaf. The cell densities were normalized based on OD_660_ prior to obtaining bacterial absorbance spectra.

In order to identify which wavelengths could potentially be used by the bacteria, but would not be used by plants, absorption spectra of leaves and of *R. palustris* TIE-1 were recorded (Figure 1). While the absorption of photons by leaves drops to zero beyond ∼750 nm, the bacteria still show some absorbance in the 750–900 nm region. These spectra are similar to what has already been described for plant leaves (McCree, 1971; Noda et al., 2014; Nelson and Bugbee, 2015) and *R. palustris* (Hayashi et al., 1982; Gall and Robert, 1999; Qi et al., 2017). The absorption spectra for the bacteria were similar when grown with white, 665, 735, or 850 nm LED light (Supplementary Figure 2). The two peaks at ∼800 and ∼870 nm in *R. palustris* have been ascribed to the two bacteriochlorophylls in purple non-sulfur bacteria (Hayashi et al., 1982; Gall and Robert, 1999). Thus, while these longer wavelengths are not used by plants, they might be used by the bacteria, a phenomenon that would be useful in cases where plants and the bacteria are grown in a closed system that has limited access to light.

It is noted that the intensities of the bacteriochlorophyll pigments varied between the two light conditions shown here (Figure 1) and that the peak corresponding to the second bacteriochlorophyll was slightly shifted when grown under incandescent light as compared to 850 nm LED. This could be a function of either the intensity or the spectrum of light used, and in this study, it is unclear which of the two factors play a role in causing the observed variations.

### Effect of Wavelength on *R. palustris* TIE-1 Growth

While IR photons have been used to culture purple non-sulfur photosynthetic bacteria in the past (Kuo et al., 2012; Zhou et al., 2014; Qi et al., 2017; Xiao, 2017), the effects of different wavelengths on the growth of the bacteria was not well described. The earlier studies quantified intensity as lux (Kuo et al., 2012; Zhou et al., 2014; Qi et al., 2017) or W m^–2^ (Xiao, 2017), which weight the photons in favor of human vision and total energy, respectively. Based on the Stark–Einstein law, intensity should be based on the number of photons delivered to the culture. ATP production by photophosphorylation is related to the number of absorbed photons and so normalizing intensities based on photon flux density is essential to understanding the effects of different wavelengths on bacterial growth.

**FIGURE 2:**
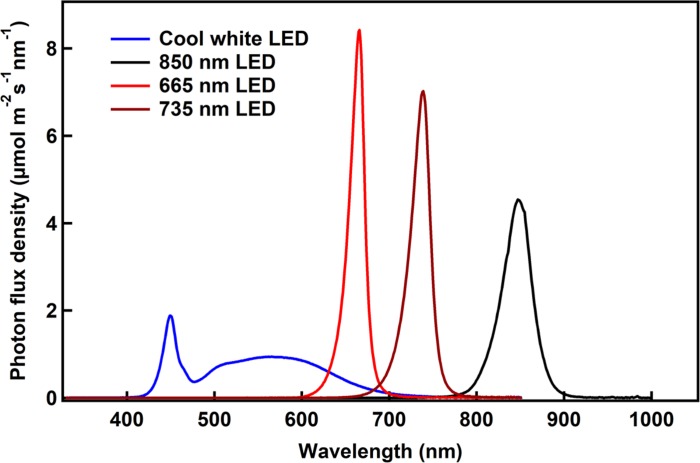
The spectroradiometric traces of the different LED lights used in the experiments are shown. The photon flux density as calculated as the area under the curve is ∼200 μmol m^–2^ s^–1^ for all the conditions.

Light emitting diodes are a new technology that can provide light of narrow wavelength bands, enabling us to study the effects of specific wavelengths on bacterial growth. Longer wavelengths were chosen to facilitate identification of wavelengths that would be minimally used by plants, but may be used to grow bacteria. Spectra of the LEDs used in these studies showed that the red, far-red and IR sources provided a narrow spectrum, with peak wavelengths of 665, 735, and 850 nm (Figure 2). The cool white LED provided a broad spectrum light source including the lower wavelengths (Figure 2). It was not possible to compare the growth rates under LED lights to that under incandescent light, which is typically used to grow these bacteria, as it was not feasible to obtain a complete spectrum of the incandescent light due to the limited range of the spectroradiometer used. So calculation of the total number of photons delivered to the bacteria was not possible.

**FIGURE 3:**
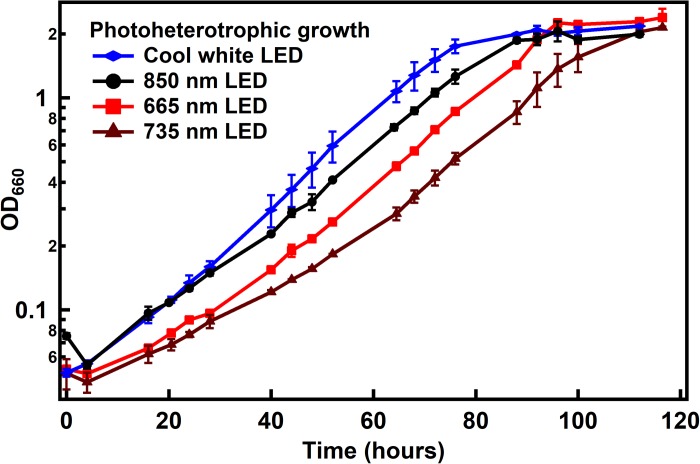
Growth of *R. palustris* with different light sources under photoheterotrophic conditions. Doubling times were found to be ∼16 h under all light conditions except with 850 nm LED (∼20 h). When calculated as specific growth rate, it was found to be 0.5 h^–1^ for all light conditions except under 735 nm LED (0.4 h^–1^).

*Rhodopseudomonas palustris* TIE-1 was cultured under photoheterotrophic conditions with acetate and ammonium sulfate as carbon and nitrogen sources, respectively. Interestingly, it was found that the doubling time of the bacteria was the same for three wavelengths used in this study – broad-spectrum cool white, 665 or 735 nm (∼16 h) – and was only slightly longer under 850 nm (∼20 h) (Figure 3). This difference in growth rate was not due to higher absorption of IR light by medium components compared to the lower wavelengths since absorption of 400–900 nm wavelengths by the medium was negligible over the path length of the culture vial (data not shown). Since bacterial growth is exponential, the growth rates were also calculated based on the equation for exponential growth after 40 h until the end of log phase for each growth condition (see Supplementary Material for more detailed calculations of specific growth rate and doubling times). The specific growth rates were very similar under all wavelength conditions (0.05 h^–1^), while marginally slower under 735 nm LED (0.04 h^–1^). It is unclear if this difference in specific growth rate is due to the error within the measurement or due to differences in the light conditions tested.

### Growth Under Photoautotrophic, Diazotrophic Conditions Using IR Radiation

**FIGURE 4:**
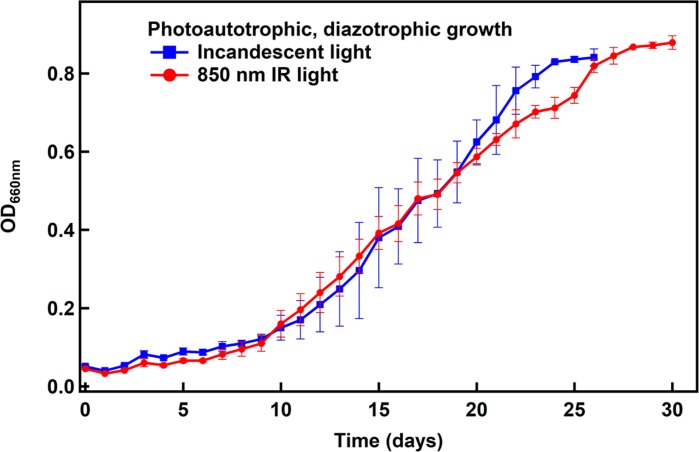
Photoautotrophic, diazotrophic growth of *R. palustris* TIE-1 under non-electrochemical conditions. The doubling time was found to be ∼4 days under both light conditions.

N_2_ fixation by *R. palustris* is an energy intensive process, and simultaneous CO_2_ fixation requires additional energy. Given the lower energy of light beyond 800 nm, it was important to study if IR light would support growth similar to the standard light source (incandescent light) when grown under N_2_- and CO_2_-fixing conditions. *R. palustris* TIE-1 was cultured under photoautotrophic, diazotrophic conditions using either 850 nm or standard incandescent light. As can be seen (Figure 4), the cells grow equally well under both conditions, revealing that 850 nm photons can support the energy demands for both CO_2_ and N_2_ fixation.

### Growth in a *R. palustris*-Electrode Hybrid System

**FIGURE 5:**
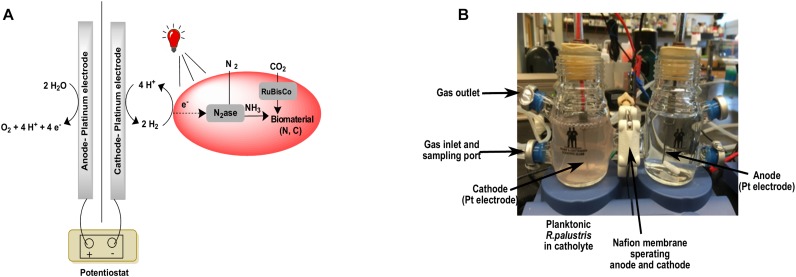
A schematic version of the bioelectrochemical system **(A)** and the actual bioelectrochemical setup used in the experiment **(B)**.

**FIGURE 6:**
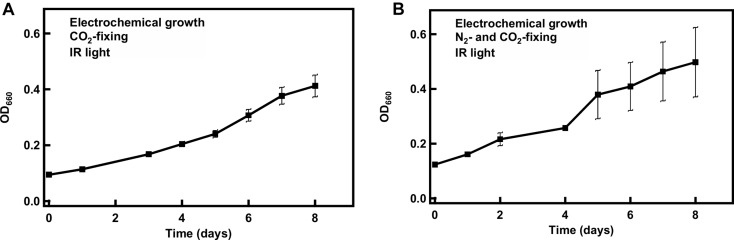
Growth of *R. palustris* TIE-1 in a hybrid system under CO_2_-fixing **(A)** or N_2_- and CO_2_-fixing **(B)** conditions. The doubling time was ∼4 days under both growth conditions.

After revealing that 850 nm photons could be used to support *R. palustris* TIE-1 grown under CO_2_ and N_2_ fixation conditions in the presence of H_2_, cells were grown in the cathodic side of an electrolysis cell with *in situ* generation of H_2_. Electrolysis of water can provide a sustainable and regular supply of H_2_ for bacterial growth (Figure 5). The working electrode was maintained at around −3 V vs. the counter electrode using a potentiostat. This potential difference was chosen in order to provide the currents necessary to produce the amount of H_2_ that would support bacterial growth. The experiments were performed at constant potential and not constant current as this was beyond the capability of the potentiostat used. H_2_ evolution was observed as bubbles formed at the platinum cathode. The bacteria grew under CO_2_-fixing or N_2_- and CO_2_-fixing conditions in this hybrid system using 850 nm LED as the light source and the electrocatalytically produced H_2_ as the electron source (Figure 6). The average doubling time was ∼4 days under both growth conditions, which was similar to growth under non-electrochemical, N_2_- and CO_2_-fixing conditions. No growth was observed over a period of 7 days when light or current was turned off (data not shown). Intriguingly, in the hybrid system, no lag phase was observed (Figure 6), although a significant lag phase (∼10 days) was observed under non-electrochemical conditions (Figure 4).

The extracellular medium was tested for the presence of fixed nitrogen to confirm that growth of the bacteria in the hybrid system with NF medium was indeed under diazotrophic conditions. A modified *o*-phthalaldehyde fluorescence method previously described (Allison et al., 1984) was used to monitor the extracellular medium for the presence of primary amines and/or ammonia over the course of the diazotrophic bioelectrochemical growth. No signal above background was observed during the growth period (data not shown) indicating the absence of sufficient fixed nitrogen sources in the medium that could have supported their growth.

The amount of H_2_ produced per day in the hybrid was estimated using the charge passed through the system (Supplementary Figure 1). It should be noted that the actual amount of H_2_ produced might be lower than what was calculated since the electrons driven into the solution from the cathode may also be used to drive reactions other than proton reduction. Since the electrolyte used was the growth medium, composed of a number of compounds including several metal salts, it was not possible to identify which (if any) other electrochemical reactions were occurring at the electrode. Assuming an ideal situation where all the electrons are used to reduce protons, the amount of hydrogen produced was estimated to be ∼288 μmol day^–1^.

At the end of the 8 day electrochemical N_2_-, CO_2_-fixing growth, the total cell dry weight fixed was found to be 0.13 g 100 mL^–1^. Assuming that 14% of the cell dry weight is N and 50% is C, 16.25 μmol of N atoms and 67.7 μmol C atoms were fixed per day for the 100 mL bioreactor. In order to calculate the efficiency of the system, the percentage of electrons delivered by the cathode that were converted into fixed N and fixed C products was calculated. The nitrogenase reaction produces 1 H_2_ for every N_2_ reduced under ideal conditions. Knowing this minimal stoichiometry (assuming 0% recycling of H_2_ produced by the nitrogenase reaction), it was calculated that 11% of electrons were used for fixing N. If it is assumed that 100% of the H_2_ produced by nitrogenase was recycled, the faradaic efficiency for N_2_ fixation is 8.5%. Similarly, the reduction of CO_2_ by the Calvin cycle requires a minimum stoichiometry of four electrons per CO_2_ reduced. Using this assumption, it was calculated that 47% of the electrons were used for direct fixation of CO_2_. The remaining electrons delivered at the cathode may be used for other reductive reactions by the bacteria, lost as H_2_ gas to the atmosphere, or be used to electrochemically reduce other medium components. Using similar assumptions, the faradaic efficiency was calculated for the CO_2_-fixing growths and was found to be ∼38% for reduction of carbon (see Supplementary Material for the complete calculations).

## Discussion

Bacterial–electrochemical hybrid systems offer great potential for sustainable upgrading of both CO_2_ and N_2_ to higher value chemicals. The use of *R. palustris* TIE-1 in electrosynthetic systems is not new, with prior examples using Fe(II) as a mediator (Doud and Angenent, 2014; Rengasamy et al., 2018) or direct electron transfer (Bose et al., 2014; Rengasamy et al., 2018; Guzman et al., 2019; Ranaivoarisoa et al., 2019). However, there are some inconsistencies with these reports. The direct electron transfer observed by Bose et al. (2014) could be complicated by trace concentrations of iron used in the medium, as higher currents were reported with increased Fe(II) concentrations in the medium (Doud and Angenent, 2014). Even in the case of Fe(II) as a mediator, while one report suggests that soluble Fe(II) can be used as a mediator (Doud and Angenent, 2014), a different report suggests that the Fe(II) has to be immobilized before it may act as a mediator (Rengasamy et al., 2018). Given these contradictions in the literature, exploring alternative mediators like H_2_ would be useful. Here, it was demonstrated that an electrosynthesis system comprising a photoautotrophic bacterium and an electrode to generate H_2_ could be used to fix both CO_2_ and N_2_. While there are a number of diazotrophs that can use H_2_ as an electron source, only a very small subset exist that can also use light as a source of energy as well as CO_2_ as a source of carbon. Since N_2_ fixation by the bacteria is a very energy intensive process, the use of a phototroph provides a way to sustainably produce the ATP that is needed to drive N_2_ fixation.

The growth rate (doubling time) was marginally different when grown with the 850 nm LED compared to the other wavelengths tested. However, when the growth was fit to the equation of exponential growth, the differences in growth rates were negligible between the growth conditions. This suggests that the efficiency of photon capture and excitation of an electron by the photosystem is very similar between all wavelength conditions tested. It was interesting that the bacteria were able to grow just as well with 735 nm light (based on doubling times) given that they showed minimal absorbance in the 735 nm region of the spectrum (Figure 1). Thus it is possible that despite the lower absorbance in that region, the ability of the bacteria to capture and utilize the light for photophosphorylation is similar to other light conditions tested. The bacteria were also able to use 850 nm photons exclusively for growth under both photoheterotrophic, non-diazotrophic conditions as well as photoautotrophic, diazotrophic conditions with growth rates similar to standard light conditions (incandescent light). Given that the 850 nm photons would not be able to efficiently excite the bacteriochlorophyll that absorbs at 800 nm, it was interesting that no significant growth defects were observed.

The 850 nm LED was used successfully in cooperation with the electrochemical H_2_ generation to drive CO_2_- and N_2_-reduction by the bacteria with growth rates similar to what was observed non-electrochemically. The nitrogen and carbon were captured in the biomass, which can potentially be digested and used as fertilizer. Advantages of this hybrid approach over a non-electrochemical one are “*in situ*” generation of H_2_ from the electrocatalytic water splitting, which could be generated from renewable energy sources (e.g., light or wind). Additionally, other molecular mediators like neutral red and methyl viologen may be explored, which have been shown with other microorganisms as a replacement to the H_2_ evolution reaction (Liu et al., 2018). The use of these other mediators could reduce the applied potential and increase faradaic efficiencies, thereby resulting in a more energetically efficient system.

Doubling time of the bacteria in the hybrid system was similar to what was observed under non-electrochemical conditions and no lag phase was observed in the hybrid system. Under similar non-electrochemical conditions, a very significant lag phase of ∼10 days was observed. This could be due to the large quantity of H_2_ provided to the bacteria under non-electrochemical conditions (∼50% of the headspace). In comparison, the H_2_ produced on average in the electrochemical system was probably sufficient to replenish dissolved H_2_ utilized by bacteria without affecting or inhibiting their growth during the initial period. This lack of a lag phase was also observed in a similar system with *Xanthobacter* (Liu et al., 2017).

By using a phototroph, CO_2_ and N_2_ reduction was achieved in a microbial electrosynthetic system at much lower currents than previously reported. Average current maintained in this system was less than 1 mA, while current of 10–12 mA was needed to drive N_2_ fixation in a system with *Xanthobacter* (Liu et al., 2017). Thus, the use of a phototroph provides a significant advantage over non-phototrophs by providing energy from light. This is evident from the calculated faradaic efficiencies of this system for N_2_ fixation (∼8.5%) as compared to a previously reported system that used the chemolithotroph *Xanthobacter* (∼4.5%) (Liu et al., 2017). Despite the large differences in currents between the two systems, the potential difference across the electrodes were the same in both studies. This could be optimized further by improving electrolyte concentrations, reducing distances between the electrodes or using a better H_2_-evolution catalyst that is more resistant to passivation over longer durations. *R. palustris* has been found to tolerate relatively high amounts of salt, growing well with 200 mM NaCl concentration in this system. Further studies optimizing the bioelectrochemical system for salt concentration, electrode material, and reactor configuration could improve the energy efficiency of the system. Different configurations of light may also be used to improve bacterial growth and increase cell yields as has been found in another similar system (Doud and Angenent, 2014).

Another advantage in using *R. palustris* is that a genetic system for this bacteria is well established and a complete genome sequence is available (Larimer et al., 2003). Thus genetic manipulation for extracellular ammonia production could be performed to obtain ammonia rather than organic nitrogen from biomass. In fact, a mutant of *R. palustris*, which constitutively expresses nitrogenase, has been shown to generate extracellular ammonium when grown under NF conditions (Adessi et al., 2012). Although the uptake hydrogenase is non-functional in the mutant and so cannot be applied to this system, this indicates that genetic manipulation of the bacteria could force them to release ammonia into the extracellular medium which would be readily accessible. This mutant would also be useful in systems that replace H_2_ with other non-gaseous mediators like neutral red or methyl viologen. The use of glutamine synthetase inhibitors would be another viable option, as was used in the study with *Xanthobacter*, to obtain extracellular ammonia (Liu et al., 2017). Apart from ammonia as the main product from N_2_ fixation, this system may also be potentially applied to drive CO_2_ reduction to valuable carbon compounds. Given the established genetic system in the bacteria, metabolic engineering of the bacteria can be performed to generate mutants that would be able to convert CO_2_ into precursors for bioplastics (Ranaivoarisoa et al., 2019), pharmaceuticals, or biofuels. For example, earlier studies showed that *R. palustris* can produce methane from CO_2_ in a single enzymatic step catalyzed by the nitrogenase enzyme (Fixen et al., 2016). When combined with the bacterial-electrode hybrid system, it is possible to develop an energy sustainable system for *in situ* production of both fixed C and N.

## Author Contributions

MS, RL, LS, SE, BB, and PL conceived the experiments. MS, RL, PK, and SZ carried out the experiments. MS wrote the manuscript with inputs from all authors.

## Conflict of Interest Statement

The authors declare that the research was conducted in the absence of any commercial or financial relationships that could be construed as a potential conflict of interest.
