# Supplementary material for: Phototrophic N2 and CO2 Fixation Using a Rhodopseudomonas palustris-H2 Mediated Electrochemical System With Infrared Photons
Source: Front Microbiol. 2019 Aug 14;10:1817. doi: 10.3389/fmicb.2019.01817 (PMC6705187; doi:10.3389/fmicb.2019.01817)
Supplement: Supplementary file 1 [file Data_Sheet_1.pdf]

# Supplementary Material

## Calculations of faradaic efficiencies

$$\text{Faradaic efficiency} = \frac{\text{Amount of N or C fixed} \times \text{Number of electrons needed}}{\text{Total number of electrons delivered at cathode}} \times 100$$

## $N_2$-, $CO_2$-fixing bioelectrochemical cell

At OD ~0.9, cell dry weight = 300 mg/L.
Therefore, at an OD of ~ 0.12 (OD at the start of the experiment), cell dry weight = 40 mg/L
At OD ~0.5 (OD at the end of 8 days in 100 mL bioelectrochemical cell), cell dry weight = 170 mg/L
Cell dry weight fixed in 8 days = 130 mg/L

**Fixed N:**
Assuming 14% of cell dry weight is N, weight of fixed N after 8 days = 1.82 mg/100 mL
Average rate of fixed N production = 0.23 mg/100 mL/day = 16.25 µmol N/100 mL/day
Number of electrons delivered at cathode = 576 µmol/100 mL/day

Number of electrons needed to fix one N atom (Assuming 0% recycling of $H_2$ produced by nitrogenase) = 4
Faradaic efficiency of N fixation = 11.3%

Number of electrons needed to fix one N atom (assuming 100% recycling of $H_2$ produced by nitrogenase) = 3
Faradaic efficiency of N fixation = 8.5%

**Fixed C**

Assuming 50% of cell dry weight is C, weight of C fixed after 8 days = 6.5 mg/100 mL
Average rate of fixed C production = 0.8 mg/100 mL/day = 67.7 µmol C/100 mL/day
Number of electrons delivered at cathode = 576 µmol/100 mL/day
Number of electrons needed to fix one C atom = 4 (2 NADPH, and hence 4 electrons per $CO_2$ needed for the Calvin cycle only; not considering electrons needed for further reduction to sugars or fatty acids)

Faradaic efficiency of C fixation = 47%

**$CO_2$-fixing bioelectrochemical cell**

At OD ~0.9, cell dry weight = 300 mg/L.
Therefore, at OD ~0.095 (OD at the start of the experiment), cell dry weight = 31.7 mg/L
At OD ~0.41 (OD at the end of 8 days in 100 mL bioelectrochemical cell), cell dry weight = ~137 mg/L
Cell dry weight fixed after 8 days = 105 mg/L

**Fixed C**

Assuming 50% of cell dry weight is C, weight of C fixed after 8 days = 5.26 mg/100 mL
Average rate of fixed C production = 0.66 mg/100 mL/day = 54.8 µmol C/100 mL/day
Number of electrons delivered at cathode = 577 µmol/100 mL/day
Number of electrons needed to fix one C atom = 4 (2 NADPH, and hence 4 electrons per $CO_2$ needed for the Calvin cycle only; not considering electrons needed for further reduction to sugars or fatty acids)

Faradaic efficiency of C fixation = 38%

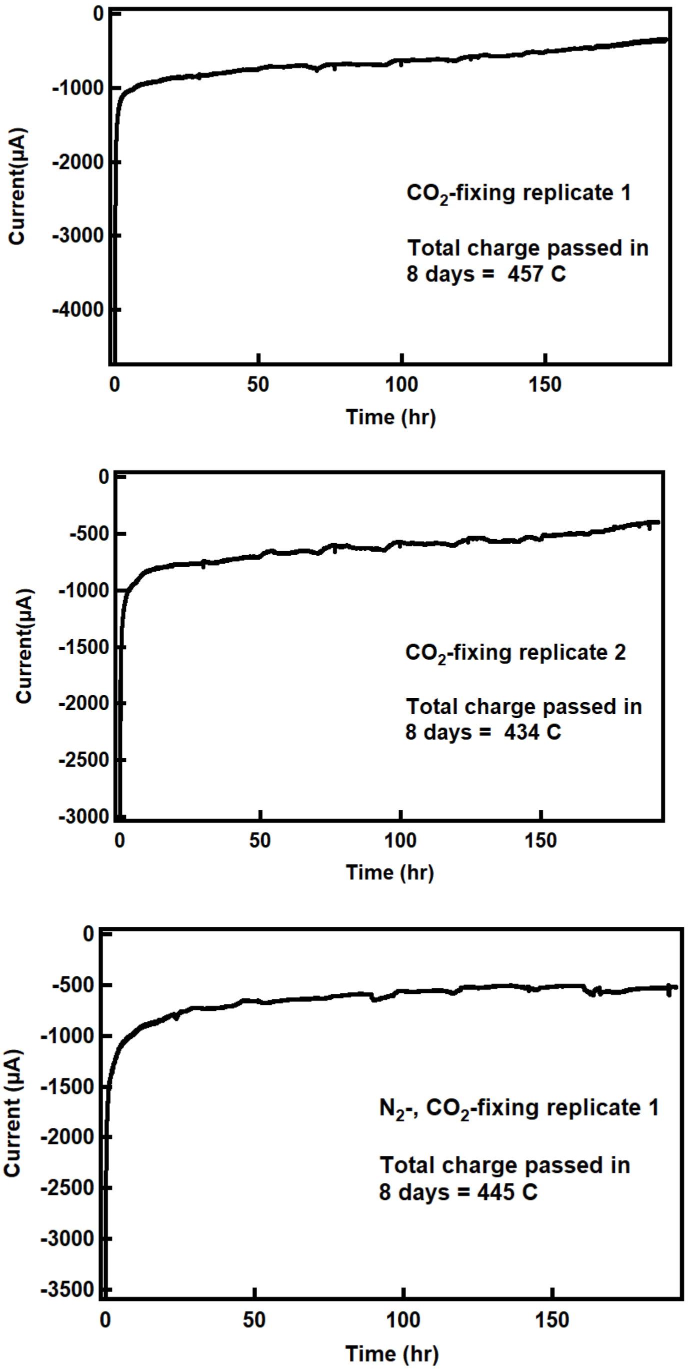


**Supplementary Figure 1:** Current passed through the bioelectrochemical system under different growth conditions. All the bioelectrochemical growths were performed at a potential difference of -3 V with respect to the counter electrode (~ -1.4 V vs Ag/AgCl reference) with 16-guage platinum wires as the cathode and anode. The electrolytes were not stirred under $CO_2$-fixing conditions, but were stirred under $N_2$- and $CO_2$-fixing conditions to allow for higher currents that were necessary to support bacterial growth under $N_2$-fixing conditions.

**Whole cell absorbance spectra of bacteria grown under different light conditions**

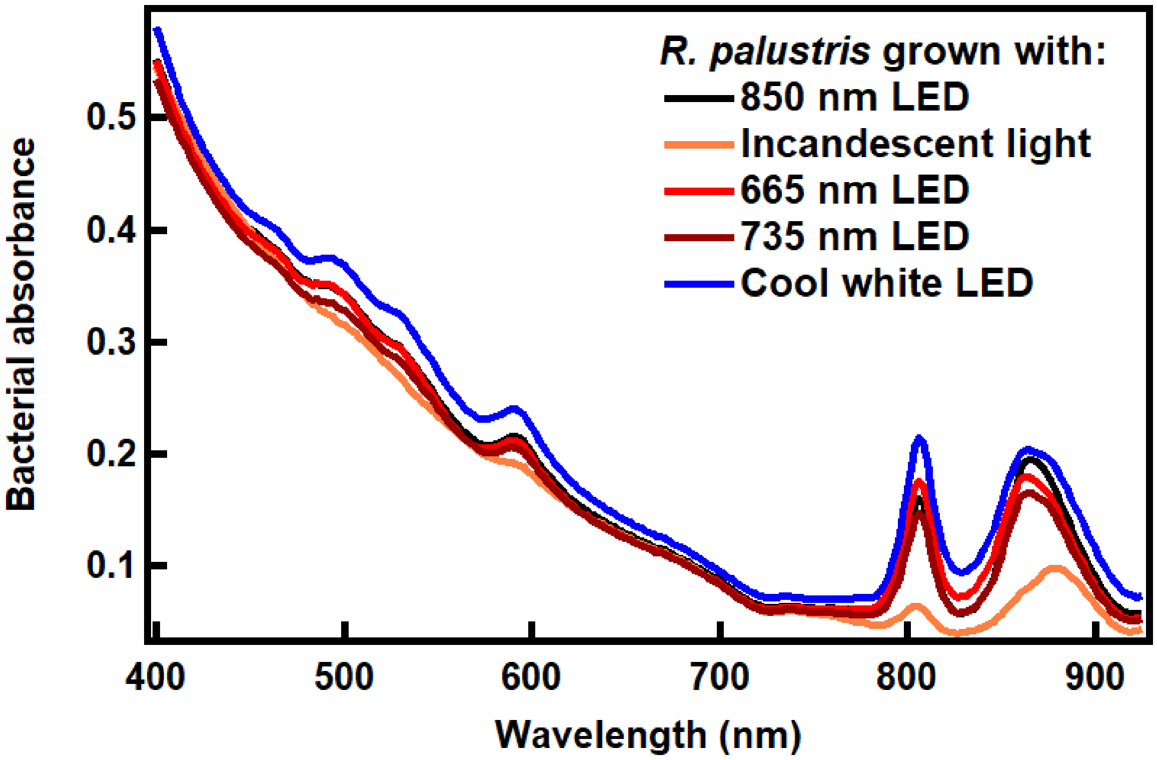


**Supplementary Figure 2:** Whole cell absorbance spectra of *R. palustris* TIE-1 grown under different light conditions. The cell densities were normalised by the $OD_{660}$.

**Calculation of doubling time**

Doubling times were calculated from the average OD measurements. The data was plotted in Microsoft Excel and the linear region was identified by fitting different time ranges to a linear trendline that would give an $R^2$ value of 0.95 or greater (**Supplementary figure 3**). Within the identified linear phase, the time taken for one doubling of the bacteria was identified and reported as the doubling time. The doubling times are summarized in **supplementary table 1**.

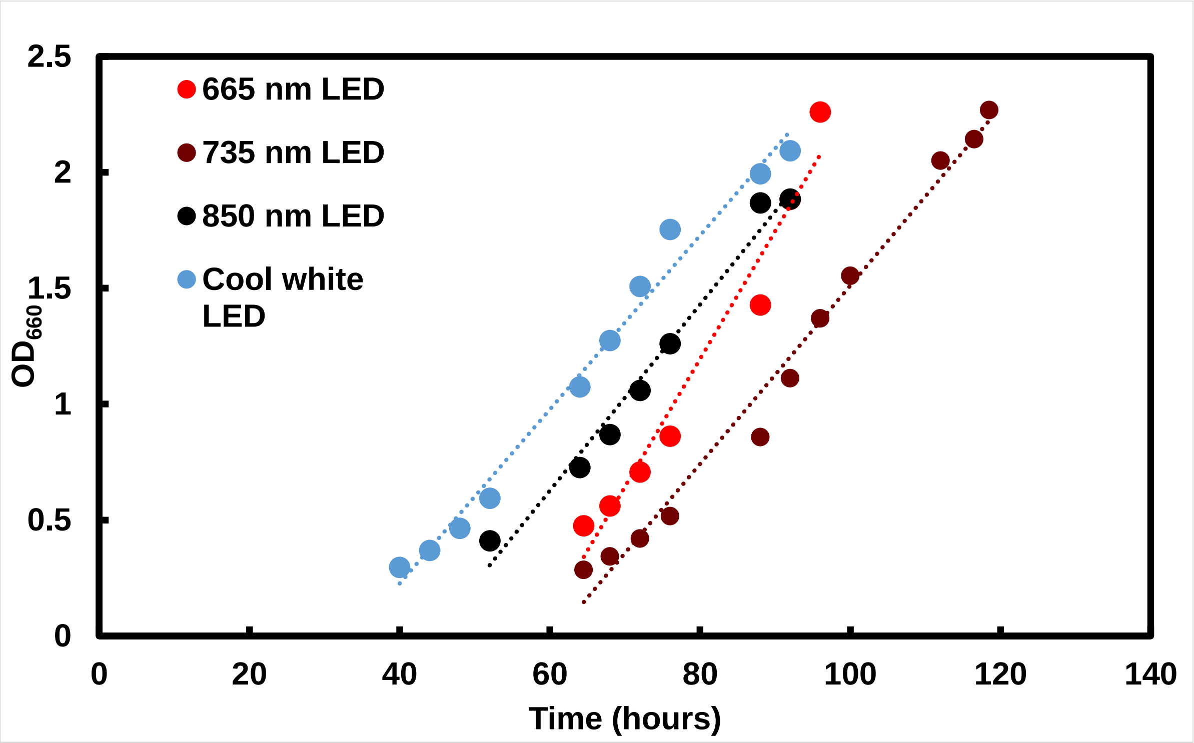


**Supplementary Figure 3:** Regions of linear growth identified for the four different light conditions identified by fitting the data to a linear trendline on Microsoft Excel 2016 and selecting the range that had the highest $R^2$ value. All $R^2$ values for the given range were above 0.95.

**<u>Calculation of specific growth rate</u>**

$$\text{Specific growth rate} = \frac{\ln \frac{A_1}{A_0}}{t_1 - t_0}$$

Where $A_0$, $A_1$ are absorbances at times $t_0$ and $t_1$ respectively.

The region of exponential growth was identified by fitting the data to an exponential curve on Microsoft Excel 2016 and identifying the data range that provided the highest $R^2$ value. (**Supplementary figure 4**). The first 40 hours was ignored for all wavelength conditions as this most likely represented the lag phase of bacterial growth. The specific growth rate identified was the average of the growth rates during the identified intervals for each growth curve. The identified growth rates are summarized in **supplementary table 1**.

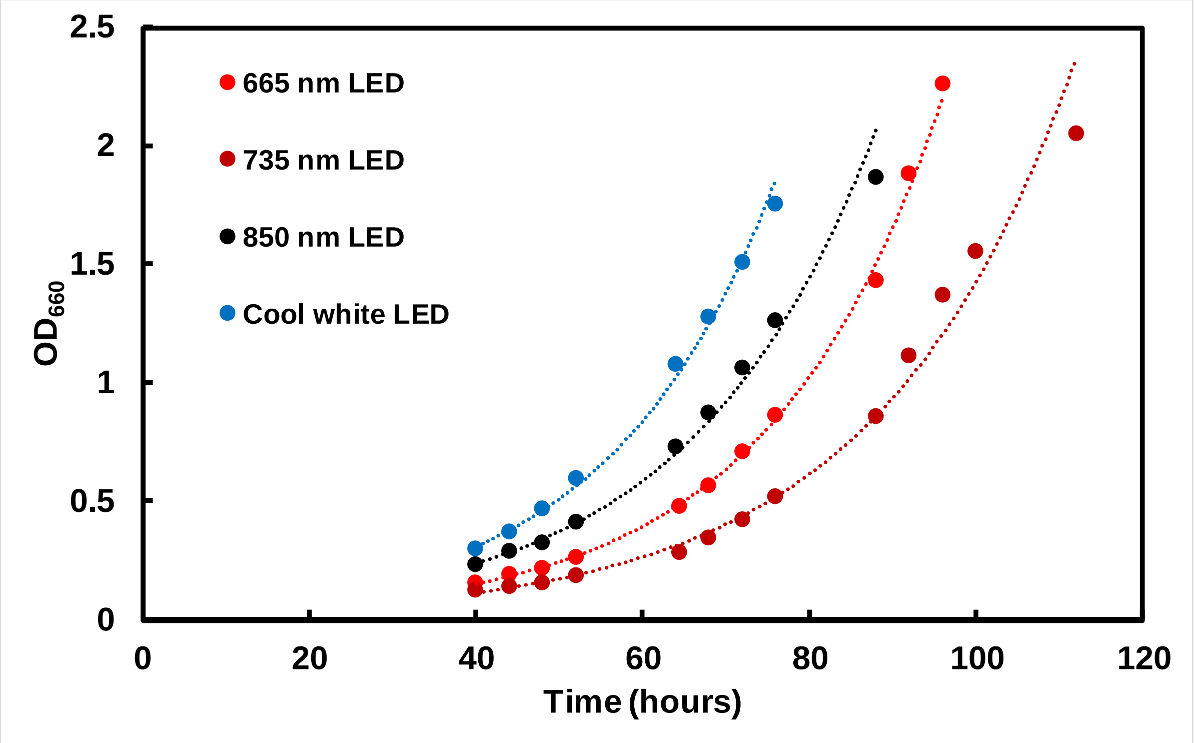


**Supplementary Figure 4:** Regions of exponential growth identified for the four different light conditions identified by fitting the data to an exponential curve on Microsoft Excel 2016 and selecting the range that had the highest $R^2$ value. All $R^2$ values for the given range were above 0.99.

**Supplementary table 1:** Summary of growth rates calculated for growth with different wavelengths of light

| **Wavelength condition** | Cool white LED | 850 nm LED | 665 nm LED | 735 nm LED |
|---|---|---|---|---|
| **Doubling time (h)** | 16 | 20 | 16 | 16 |
| **Specific growth rate ($h^{-1}$)** | 0.05 | 0.05 | 0.05 | 0.04 |
